# Recent update in diagnosis and treatment of human pythiosis

**DOI:** 10.7717/peerj.8555

**Published:** 2020-02-20

**Authors:** Maria Nina Chitasombat, Passara Jongkhajornpong, Kaevalin Lekhanont, Theerapong Krajaejun

**Affiliations:** 1 Division of Infectious Disease, Department of Medicine, Faculty of Medicine, Ramathibodi Hospital, Mahidol University, Bangkok, Thailand; 2 Department of Ophthalmology, Faculty of Medicine, Ramathibodi Hospital, Mahidol University, Bangkok, Thailand; 3 Department of Pathology, Faculty of Medicine, Ramathibodi Hospital, Mahidol University, Bangkok, Thailand

**Keywords:** *Pythium insidiosum*, Pythiosis, Diagnosis, Treatment, Management

## Abstract

Human pythiosis is an infectious condition with high morbidity and mortality. The causative agent is the oomycete microorganism *Pythium insidiosum*. The pathogen inhabits ubiquitously in a wet environment, and direct exposure to the pathogen initiates the infection. Most patients with pythiosis require surgical removal of the affected organ, and many patients die from the disease. Awareness of pythiosis among healthcare personnel is increasing. In this review, we summarized and updated information on the diagnosis and treatment of human pythiosis. Vascular and ocular pythiosis are common clinical manifestations. Recognition of the typical clinical features of pythiosis is essential for early diagnosis. The definitive diagnosis of the disease requires laboratory testing, such as microbiological, serological, molecular, and proteomic assays. In vascular pythiosis, surgical intervention to achieve the organism-free margin of the affected tissue, in combination with the use of antifungal drugs and *P. insidiosum* immunotherapy, remains the recommended treatment. Ocular pythiosis is a serious condition and earliest therapeutic penetrating keratoplasty with wide surgical margin is the mainstay treatment. Thorough clinical assessment is essential in all patients to evaluate the treatment response and detect an early sign of the disease recurrence. In conclusion, early diagnosis and proper management are the keys to an optimal outcome of the patients with pythiosis.

## Introduction

Human pythiosis is a life-threatening infectious condition exhibiting high morbidity and mortality, as most patients require surgical removal of the affected organ (Thianprasit, Chaiprasert & Imwidthaya, 1996; Krajaejun et al., 2006b; Gaastra et al., 2010). The causative agent, *Pythium insidiosum*, is a member of the unique group of aquatic fungus-like microorganisms, called oomycetes (De Cock et al., 1987), which is a common inhabitant of wet environments (Supabandhu et al., 2008; Vanittanakom et al., 2014; Presser & Goss, 2015). Direct exposure to its infectious form, zoospore, can initiate infection (Mendoza, Hernandez & Ajello, 1993). With an increase in incidence, especially from the tropical, subtropical, and temperate countries, such as Thailand (Krajaejun et al., 2006b; Lekhanont et al., 2009; Permpalung et al., 2015; Lelievre et al., 2015; Anutarapongpan et al., 2018; Worasilchai et al., 2018; Permpalung et al., 2019), India (Bagga et al., 2018; Chatterjee & Agrawal, 2018; Rathi et al., 2018; Hasika et al., 2019; Agarwal et al., 2019; Appavu, Prajna & Rajapandian, 2019), Malaysia (Badenoch et al., 2001), China (He et al., 2016), Japan (Maeno et al., 2019), Australia (Badenoch et al., 2009), New Zealand (Murdoch & Parr, 1997), Spain (Bernheim et al., 2019), Israel (Tanhehco et al., 2011; Barequet, Lavinsky & Rosner, 2013), Columbia (Ros Castellar et al., 2017), Brazil (Bosco Sde et al., 2005), Costa Rica (Neufeld et al., 2018), Jamaica (Pan et al., 2014), and the United States (Shenep et al., 1998), there has been an increase in awareness of pythiosis among healthcare personnel.

The major challenges in clinical management of pythiosis are the unavailability of established diagnostic tests and effective therapeutic modalities. Prompt diagnosis and proper treatment are critical for ensuring a more favorable prognosis. In this review, we compiled recent information on the diagnosis and treatment of human pythiosis, reported in the literature by the healthcare professions who share their in-depth experiences of the disease.

## Survey methodology

**Figure 1:**
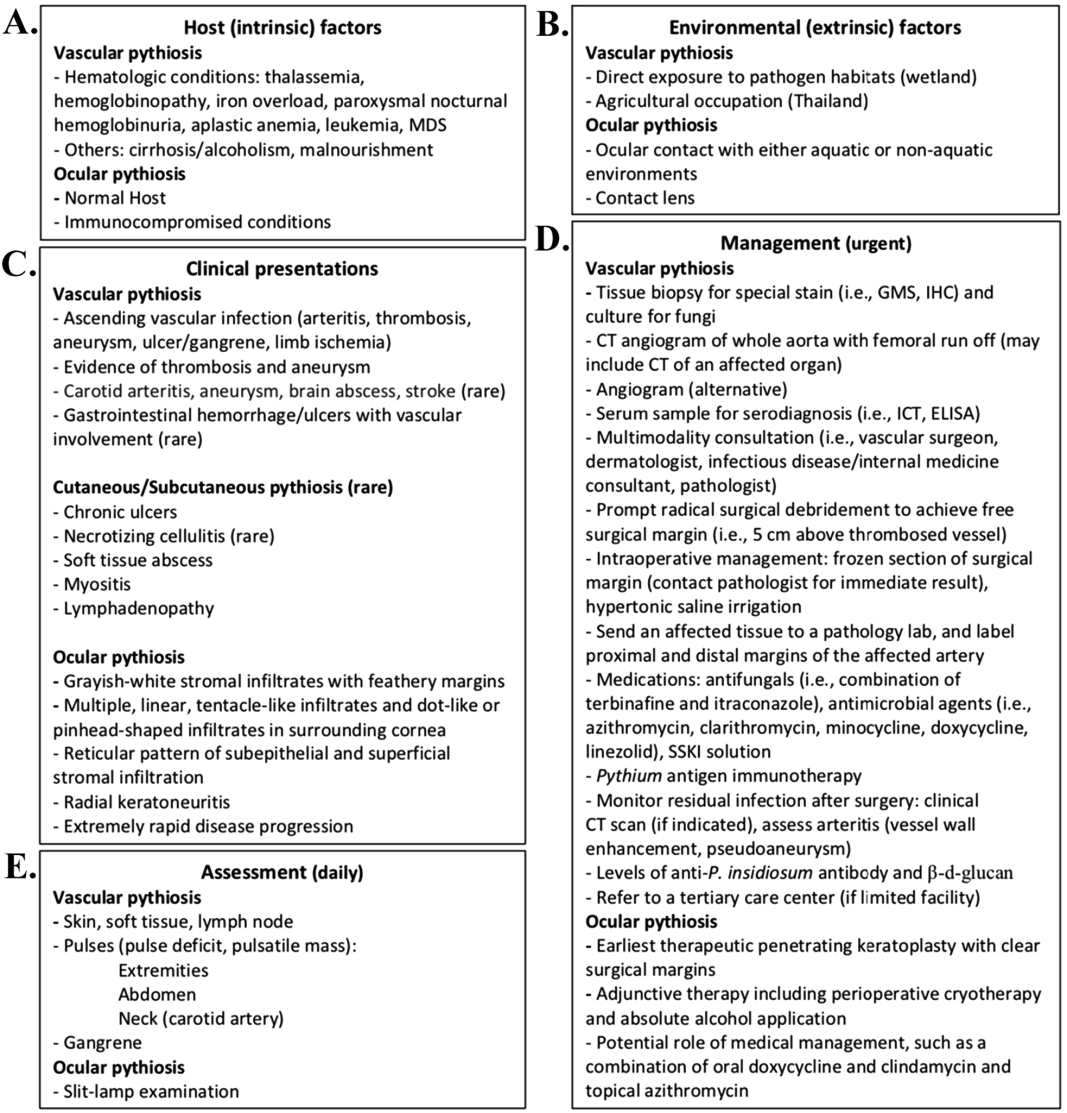
Essential information for the diagnosis and management of human pythiosis. Information on host factor (A), environmental factor (B), and clinical presentation (C) provide clinical clues of pythiosis. Once pythiosis is suspected, definitive diagnosis and proper treatment are urgent and essential for controlling infection (D). During treatment, clinical assessment of the patient with pythiosis should be performed daily (E). Abbreviations: GMS; Gomori-Methenamine Silver stain; IHC, immunohistochemistry; ICT, Immunochromatographic test; ELISA, Enzyme-linked immunosorbent assay; SSKI, Saturated solution of potassium iodide.

This study was approved by the Committee for Research, Faculty of Medicine Ramathibodi Hospital, Mahidol University (approval number: MURA2019/740). Our team, as healthcare professions in the fields of infectious diseases, ophthalmology, and pathology, performed a literature search using the PubMed database (https://www.ncbi.nlm.nih.gov/pubmed/). Based on the keywords, ‘pythiosis’ and ‘*Pythium insidiosum*’ articles extracted were summarized to identify clinical information that was relevant to diagnosis and management of human pythiosis and grouped into sections (i.e., clinical features, predisposing factors, laboratory diagnosis, management and follow-up, and clinical outcomes) and key notes (Fig. 1).

## Clinical features of pythiosis

Human pythiosis exhibits heterogenous clinical manifestations (Krajaejun et al., 2006b; Sathapatayavongs et al., 1989). Most patients present with symptoms and signs that are associated with the *P. insidiosum* infection of the medium–large artery (called vascular pythiosis) and the eye (called ocular pythiosis) (Krajaejun et al., 2006b). Unusual features of the disease include necrotizing cellulitis, deep neck abscesses, carotid aneurysm, meningitis, brain abscesses, cerebral septic emboli, and disseminated infection (Krajaejun et al., 2006b; Chitasombat et al., 2018a).

### Vascular pythiosis

**Figure 2:**
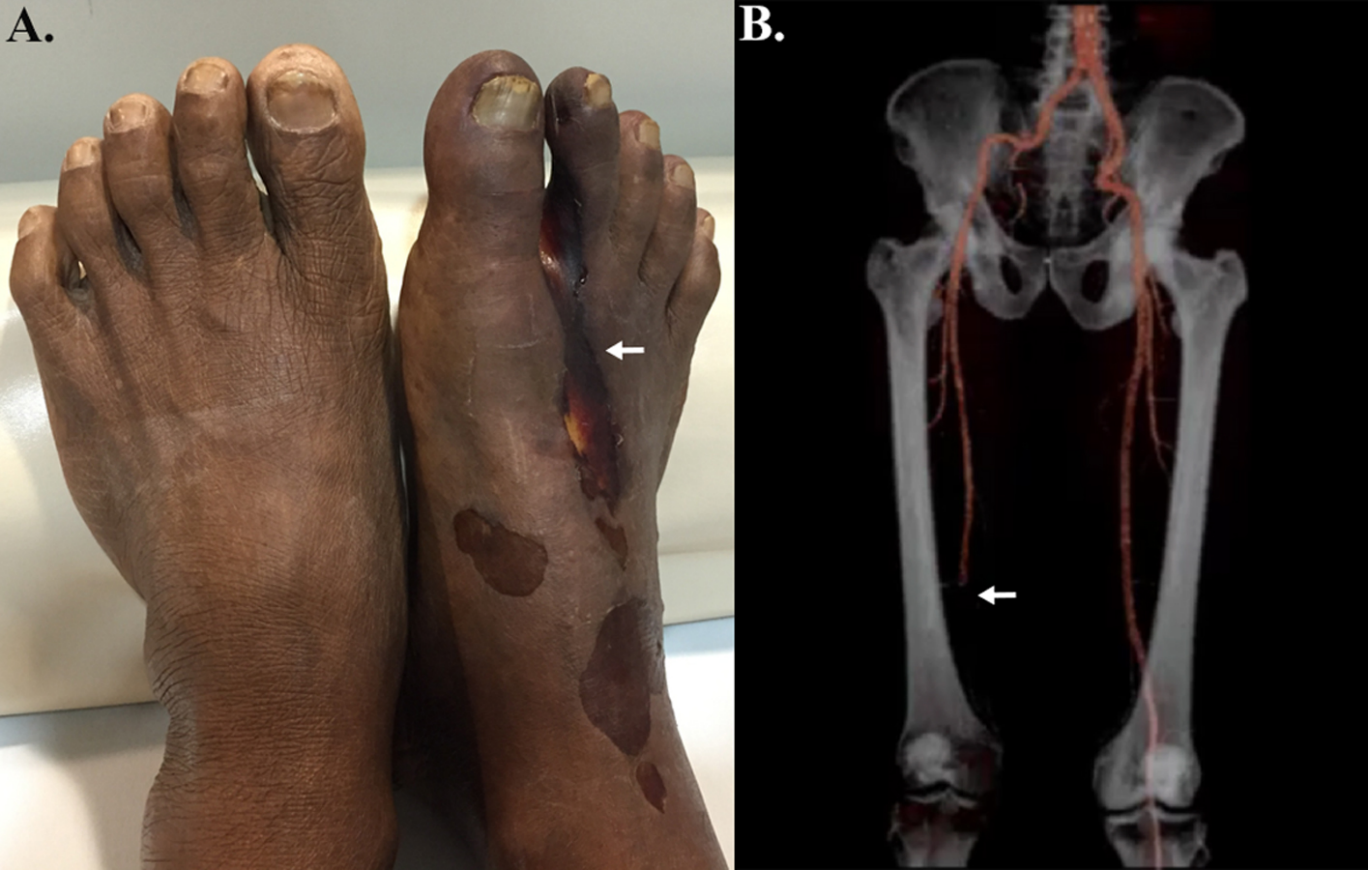
Clinical features of vascular pythiosis. (**A**) Gangrenous ulcer of the right foot (arrow); (**B**) Peripheral run-off computerized tomography angiography shows total occlusion of the right proximal-to-distal popliteal artery (arrow). (Photo credit: Patawee Boontanondha, M.D.).

Vascular pythiosis usually affects the arteries of lower extremities (Fig. 2). Skin is likely a portal of entry where the zoospore of *P. insidiosum* attaches and germinates to invade arteries and surrounding tissue. Typical patients have an underlying hematological disorder and an agriculture-related occupation. Most patients present with chronic non-healing skin lesions, arterial insufficiency syndrome (i.e., intermittent claudication, paresthesia, absence of arterial pulse, and evidence of arteritis or thrombosis), gangrenous ulcer, and pulsatile mass (i.e., a groin mass of the iliac or femoral aneurysm, and an abdominal mass of aortic aneurysm) (Krajaejun et al., 2006b; Permpalung et al., 2015; Reanpang et al., 2015; Sermsathanasawadi et al., 2016). Skin changes (i.e., color and texture), as the sequelae of arterial insufficiency, can be observed in patients with vascular pythiosis. The infection usually ascends to a proximal arterial issue, which leads to thrombosis, arterial occlusion, and aneurysm (ruptured aneurysm results in mortality). Vascular pythiosis of the upper extremities has been occasionally reported (Worasilchai et al., 2018; Khunkhet, Rattanakaemakorn & Rajatanavin, 2015). There are only a few cases of carotid artery involvement, an extremely rare but potentially fatal condition that results in meningitis, cerebral septic emboli, brain abscesses, and death. Patients with an immunosuppressed or neutropenic condition may present with abrupt-onset infection (i.e., within days after the onset of symptoms), with or without a history of exposure to aquatic habitats (Chitasombat et al., 2018a; Hoffman, Cornish & Simonsen, 2011; Hilton et al., 2016), and develop necrotic skin lesions/cellulitis that progress to vascular infections shortly afterward (within a few days or weeks).

Early recognition of pythiosis and the use of a rapid laboratory test leads to prompt and definitive therapy. Laboratory investigation (see the “Laboratory diagnosis” section) is essential for the identification of the *P. insidiosum* infection. Specimen handling is a critical step to enhance the success rate of the laboratory investigation. Specimens such as blood clot, pus, and vessel tissue (at the site of thrombosed vessels and proximal margin), should be sent, without formalin preservation, to a clinical microbiology laboratory for culture identification, staining, and/or molecular testing. Resected vessel and soft tissue specimen sent for histological examination should be labelled—proximal or distal—in order to identify the disease-free surgical margin by Grocott-Gömöri methenamine silver (GMS) staining (Sermsathanasawadi et al., 2016). Further immunohistochemistry staining for *P. insidiosum* is helpful to confirm the diagnosis, especially if the standard microbiological culture is not available or fails to identify the pathogen (Inkomlue et al., 2016; Keeratijarut et al., 2009).

### Ocular pythiosis

Patients with ocular pythiosis (also known as *Pythium* keratitis) usually have a history of predisposing factors (see the “Predisposing factors” section), and present with ocular pain, irritation, photophobia, decreased visual acuity, conjunctival redness, and eyelid swelling, similar to another microbial keratitis. Clinical onset of ocular pythiosis could range from two days to over a month. *Pythium* keratitis is often clinically indistinguishable from fungal keratitis, because the causative agents share some clinical features (such as, the presence of grayish-white stromal infiltrates with feathery margins on slit-lamp biomicroscopy) and microscopic findings (such as, septate linear branching structures in a corneal scraping sample). However, the natural history and clinical clues at the margin or surrounding areas of the main infiltrates would help to differentiate *Pythium* keratitis from fungal keratitis.

**Figure 3:**
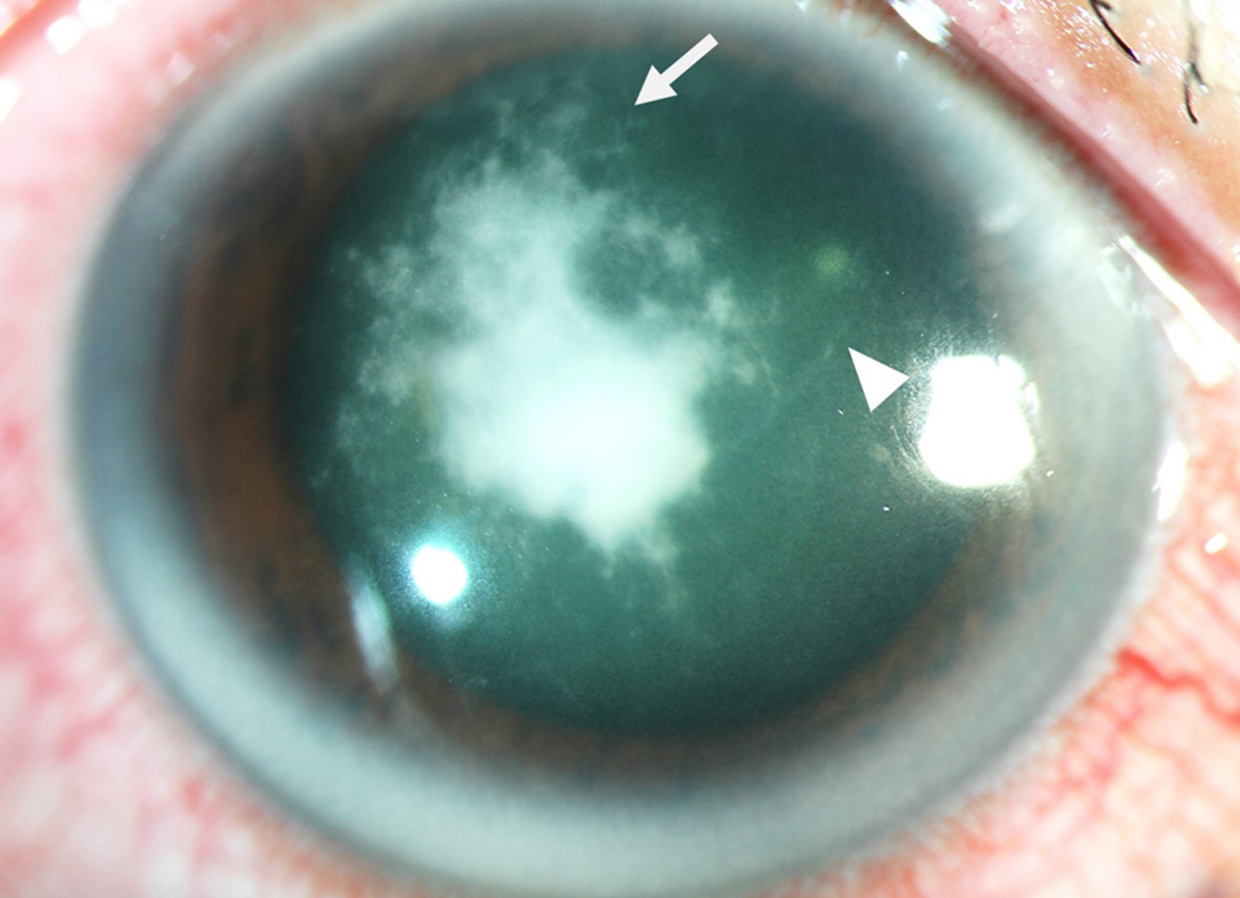
Clinical features of ocular pythiosis. Slit-lamp photograph of the infected cornea demonstrates central, dense, grayish-white infiltrates with tentacle-like extensions (arrowhead) and subepithelial dot-like infiltrates radiating in a reticular pattern from the lesion (arrow). (Photo credit: Passara Jongkhajornpong, M.D.).

Patients with *Pythium* keratitis usually present with typical clinical manifestations, including multiple, linear, tentacle-like infiltrates and dot-like or pinhead-shaped infiltrates, involving the subepithelial, anterior stromal, and midstromal layers in surrounding cornea and radiating in a reticular pattern from the central area of the lesion towards the limbus (Fig. 3) (Lekhanont et al., 2009; Lelievre et al., 2015; Bagga et al., 2018; Chatterjee & Agrawal, 2018; Agarwal et al., 2019; He et al., 2016; Thanathanee et al., 2013; Sharma et al., 2015; Agarwal et al., 2018). Radial keratoneuritis has been observed in some cases (Lekhanont et al., 2009; He et al., 2016). Corneal lesions should be closely monitored, as the typical features of *Pythium* keratitis may develop during the course of the disease (Chatterjee & Agrawal, 2018). In advance disease, these clinical clues may be obscured by dense corneal stromal infiltration (Anutarapongpan et al., 2018; Badenoch et al., 2001). The rapid and progressive nature of ocular pythiosis, despite intensive antimicrobial treatment, distinguishes *Pythium* keratitis from other fungal infections. An extensive and aggressive infiltration of *P . insidiosum* could result in corneal perforation, anterior chamber, limbal and scleral invasions, or endophthalmitis in a few days or weeks (Lekhanont et al., 2009; Lelievre et al., 2015; Rathi et al., 2018; Agarwal et al., 2019; Badenoch et al., 2001; He et al., 2016; Maeno et al., 2019; Ros Castellar et al., 2017; Thanathanee et al., 2013; Sharma et al., 2015; Agarwal et al., 2018).

Corneal scrapings should be subjected to clinical microbiology examination (i.e., fungal culture, polymerase chain reaction (PCR), and DNA sequencing; see the “Laboratory diagnosis” section) to establish a definitive diagnosis. An ocular specimen is usually obtained in a minimal amount, which could lead to a false-negative result by such laboratory tests. Serological tests are not useful because the serum anti-*P. insidiosum* antibody in patients with *Pythium* keratitis is usually undetectable (Krajaejun et al., 2006b; Permpalung et al., 2019; Krajaejun et al., 2009; Jindayok et al., 2009). Repeated corneal cultures or combining specimens, including corneal scrapings and corneal buttons, could increase the chance of a positive result (Hasika et al., 2019).

*In vivo* confocal microscopy (IVCM) is an emerging, non-invasive, real-time imaging technique that enables morphological and quantitative analysis of ocular surface microstructure in both health and disease. Clinically, IVCM is a useful diagnostic tool for the early diagnosis of microbial particularly fungal and *Acanthamoeba* keratitis (Alzubaidi et al., 2016). *P. insidiosum* hyphae can be identified using IVCM in 95% of culture- or PCR-positive patients (Anutarapongpan et al., 2018). *Pythium* keratitis manifest as hyperreflective branching structures (vary in size; 1.5–7.5 µm in diameter and 90-400 µm in length), septate linear branching structures resembling fungal filaments, and irregular filaments arranged in a sinuous, various branching pattern, forming “X”,” Y”, and” S” shapes as seen by IVCM (Lelievre et al., 2015; He et al., 2016; Tanhehco et al., 2011; Thanathanee et al., 2013). However, microbial characteristics captured by IVCM are non-specific and overlapped among *Pythium, Aspergillus*, and *Fusarium* keratitis (Anutarapongpan et al., 2018). Nevertheless, IVCM can provide rapid visualization of *Pythium* hyphae in infected cornea and is recommended when *Pythium* keratitis is suspected (Anutarapongpan et al., 2018).

### Unusual features of pythiosis

Patients with pythiosis can present with various skin manifestations, such as vesicle/bulla, skin ulcer, cellulitis, chronic swelling, painful subcutaneous lesion, infiltrative lump and ulcer on the limb (Krajaejun et al., 2006b) and rapidly progressive *P. insidiosum* infection (i.e., necrotizing cellulitis) (Krajaejun et al., 2006b; Kirzhner et al., 2015). If left untreated, cutaneous/subcutaneous infection could progress to vascular pythiosis, which can lead to amputation of the affected limb (Krajaejun et al., 2006b; Khunkhet, Rattanakaemakorn & Rajatanavin, 2015). Craniofacial infection, such as periorbital/orbital necrotizing cellulitis, is a rapid-onset clinical feature observed in children and young adults with a recent history of exposure to wetland (Krajaejun et al., 2006b; Hilton et al., 2016; Kirzhner et al., 2015; Triscott, Weedon & Cabana, 1993). Rarely, *P. insidiosum* infection can disseminate to a visceral organ and result in mortality (Krajaejun et al., 2006b), which in a patient with gastrointestinal involvement leads to upper gastrointestinal bleeding, bloody mucous stools, gastric/ileal ulcer, and peritonitis (Krajaejun et al., 2006b).

## Predisposing Factors

### Host (intrinsic) factors

Vascular pythiosis has been strongly associated with thalassemia (Krajaejun et al., 2006a; Permpalung et al., 2015; Worasilchai et al., 2018; Reanpang et al., 2015; Sermsathanasawadi et al., 2016; Thitithanyanont et al., 1998). When stimulate with *P. insidiosum* zoospore, peripheral blood mononuclear cells collected from thalassemic patients showed lower level of granulocyte-macrophage colony-stimulating factor (GM-CSF) and interferon gamma (IFN-γ) production, compared with the non-thalassemic controls (Ud-Naen et al., 2019). Other hematologic disorders that predispose individuals to vascular pythiosis include paroxysmal nocturnal hemoglobinuria, aplastic anemia, myelodysplasia, idiopathic thrombocytopenic purpura, and leukemia (Krajaejun et al., 2006b), and to a lesser extent, young age, alcoholism, malnourishment, immunosuppression, HIV infection, cancer, and neutropenia (Krajaejun et al., 2006b; Pan et al., 2014; Shenep et al., 1998; Chitasombat et al., 2018a; Hoffman, Cornish & Simonsen, 2011; Hilton et al., 2016; Kirzhner et al., 2015; Franco et al., 2010; Chitasombat et al., 2018b).

In contrast to vascular pythiosis, ocular pythiosis commonly affects healthy individuals (Krajaejun et al., 2006b; Lekhanont et al., 2009; Krajaejun et al., 2004). However, in a series reported from Northeastern Thailand, 50% of patients with *Pythium* keratitis had thalassemia hemoglobinopathy (Thanathanee et al., 2013), though the prevalence, and the heterogeneity, of thalassemia hemoglobinopathies is high among Thai people living especially in Northeastern Thailand. Thus, whether thalassemia is a predisposing factor in ocular pythiosis is not known, warranting the need for additional studies to understand the relationship between ocular pythiosis and thalassemia hemoglobinopathies. Additionally, a fulminant form of *Pythium* keratitis can be observed in immunocompromised patients (such as those with diabetes or Crohn’s disease treated with immunotherapy), which resulted in poor clinical outcomes (i.e., enucleation) (Rathi et al., 2018; Neufeld et al., 2018).

### Environmental (extrinsic) factors

*P. insidiosum* inhabits stagnant water and soil, and colonizes aquatic vegetation (Supabandhu et al., 2008; Vanittanakom et al., 2014; Mendoza, Hernandez & Ajello, 1993). Potential risk factors for pythiosis include farming exposure, direct contact with a water resource (i.e., lake, river, lagoon, swamp, or even swimming pool), and a history of travel to endemic countries, such as Thailand (Krajaejun et al., 2006b; Lelievre et al., 2015; Hasika et al., 2019; Appavu, Prajna & Rajapandian, 2019). Inappropriate hygiene of contact lens can pose a risk (Lekhanont et al., 2009; Lelievre et al., 2015; Badenoch et al., 2001; Maeno et al., 2019; Bernheim et al., 2019; Tanhehco et al., 2011; Barequet, Lavinsky & Rosner, 2013; Neufeld et al., 2018; Raghavan et al., 2018), and exposure to dust or some foreign body in their eyes (Bagga et al., 2018; Hasika et al., 2019) predisposes individuals with no agricultural activity to this infection. The incidence of pythiosis follows a seasonal trend (Hasika et al., 2019; Thanathanee et al., 2013) with outbreaks of *Pythium* keratitis occurring during the rainy season in Thailand (Thanathanee et al., 2013) and around June to September during monsoon season in South India (Hasika et al., 2019).

## Laboratory Diagnosis

Diagnosis of pythiosis is necessary to ensure prompt treatment and promote better prognosis of pythiosis patients. This requires diligent records of medical history, physical examination of the patient, and laboratory information for diagnosing definitive pythiosis using laboratory methods summarized below:

### Direct examination

Clinical specimens (i.e., purulent discharge, corneal scraping, blood clot, and pathologic tissue) obtained from a lesion are subjected to direct wet mount examination, using 10% KOH preparation (Krajaejun et al., 2006b; Mendoza, Ajello & McGinnis, 1996). Although the results obtained may not be specific, microscopic observation of broad and occasionally sparsely septate filamentous elements alerts to a further identification of the causative organism (i.e., *P. insidiosum* and some other pathogenic fungi).

### Sample handling and culture identification

Transportation of an infected patient tissue with suspected pythiosis to a clinical microbiology laboratory is a critical step for successful isolation of *P. insidiosum*. Temperature control is essential to ensure viability, and failure to isolate *P. insidiosum* is linked to storage of clinical specimen in low-temperature containers, such as icebox, refrigerator, or freezer (Brown & Roberts, 1988; Bentinck-Smith et al., 1989). Optimal growth of *P. insidiosum* occurs between 28 °C and 37 °C and extreme temperature as low as 8 °C or as high as 42 °C can completely inhibit or even kill the organism (Krajaejun et al., 2010). When immediate transportation of a culture specimen is not possible, it is recommended to store the sample in sterile distilled water while transferring to the clinical microbiology laboratory (Mendoza, Ajello & McGinnis, 1996).

*P. insidiosum* grows well on standard agar types, such as Sabouraud dextrose agar, potato dextrose agar, corn meal agar, and blood agar (Chaiprasert et al., 1990; Grooters et al., 2002). After inoculation of a viable *P. insidiosum*-containing tissue on agar of choice, a yellow-to-white or colorless-to-white flat colony can be observed within a few days (the color may depend on the culture medium used and the age of the colony), with a radial growth rate of ∼5 mm/day (De Cock et al., 1987; Krajaejun et al., 2010). Under the microscope, *P. insidiosum* appears as a right-angle branching, sparsely septate, broad (up to 10 µm in diameter) hyphae (Mendoza, Ajello & McGinnis, 1996). Zoosporangium (a sac formed at the hyphal tip that contains multiple mobile biflagellate zoospores) is a unique characteristic that differentiates *P. insidiosum* from fungi. Zoosporogenesis can be induced in an experienced laboratory by using sterile grass leaves and the induction solution, as described in details elsewhere (Chaiprasert et al., 1990; Mendoza & Prendas, 1988). Although *P. insidiosum* is a prominent pathogenic oomycete of humans, other oomycetes, such as *Lagenidium* spp. and *Pythium aphanidermatum* have also been reported, to a much lesser extent, as a human pathogen (Calvano et al., 2011; Reinprayoon et al., 2013; Farmer et al., 2015). Of late, the accurate identification of *P. insidiosum* requires immunodiagnostic, molecular, or proteomic assay (Badenoch et al., 2001; Vanittanakom et al., 2004; Intaramat et al., 2016; Krajaejun et al., 2018).

### Serological tests and biomarkers

Detection of anti-*P. insidiosum* antibody can facilitate the diagnosis of pythiosis. Several methods have been developed to detect serum *P. insidiosum*-specific antibodies. Two types of crude proteins, (i) culture filtrate antigen (CFA) and (ii) soluble antigen from broken hyphae (SABH) (Krajaejun et al., 2006a), extracted from *P. insidiosum* serve as antigens for serological tests. Immunodiffusion (ID) is a simple, cost-effective, and specific assay that is designed to observe in-gel immunoprecipitation lines generated by anti-*P. insidiosum* antibodies in patient serum and the *P. insidiosum* crude protein extract (Imwidthaya & Srimuang, 1989; Pracharktam et al., 1991). Major drawbacks of ID include poor detection sensitivity leading to a false-negative result, and longer turnaround time (∼24 h) (Krajaejun et al., 2009; Jindayok et al., 2009; Krajaejun et al., 2002; Chareonsirisuthigul et al., 2013). An enzyme-linked immunosorbent assay (ELISA) has circumvented the limitation of ID, as it shows higher sensitivity and shorter turnaround time (∼3–4 h) (Intaramat et al., 2016; Krajaejun et al., 2002; Chareonsirisuthigul et al., 2013). Western blot (WB) for immunodetection of *P. insidiosum* (Krajaejun et al., 2006a; Supabandhu et al., 2009) has been developed though its diagnostic application is limited due to the multi-step procedures involved and complexity. Hemagglutination (HA) and immunochromatographic test (ICT) are rapid and user-friendly assays that detect anti-*P. insidiosum* antibodies within 30–60 min (Krajaejun et al., 2009; Jindayok et al., 2009; Intaramat et al., 2016). However, implementation of a serodiagnostic test in a non-endemic region is challenging. Transportation of the specimen could impact the assay turnaround time.

Comparison of the above-mentioned serological assays, using a set of 37 pythiosis patients and 248 control sera, shows that ELISA (sensitivity, 100%; specificity, 100%) and ICT (sensitivity, 100%; specificity, 100%) outperform ID (sensitivity, 68%; specificity, 100%) and HA (sensitivity, 84%; specificity, 82%) (Chareonsirisuthigul et al., 2013). Evaluation of ELISA and ICT using a different set of 28 pythiosis and 80 control sera, demonstrates the greater sensitivity of ELISA compared with ICT (96% vs. 86%) (Intaramat et al., 2016). Each serological assay has its own advantages and disadvantages. Selection of an appropriate test for serodiagnosis of pythiosis depends on assay availability, experience of the laboratory personnel, preferred test performance and turnaround time. Most importantly, the serological assays described here often fail to detect anti-*P. insidiosum* antibodies in patients with ocular pythiosis (Krajaejun et al., 2009; Jindayok et al., 2009; Intaramat et al., 2016). Therefore, one should be aware of false-negative results when performing the assays on samples from an ocular patient.

During the clinical course of pythiosis, levels of anti-*P . insidiosum* antibodies can be monitored using one of the serological tests. A gradual decrease in anti-*P. insidiosum* antibody levels over the course of months, in association with clinical improvement, indicate favorable prognosis (Jindayok et al., 2009; Pracharktam et al., 1991; Krajaejun et al., 2002). In contrast, increased or sustained antibody levels may be observed after administration of the *P. insidiosum* immunotherapeutic antigen (PIA) vaccine, prepared from crude protein extracts of the pathogen, in clinically-cured pythiosis patients (Worasilchai et al., 2018; Krajaejun et al., 2002). Recently, Worasilchai et al. (2018) reported the use of serum β-d-glucan as a surrogate marker for monitoring 50 vascular pythiosis patients after a combination of medical and surgical treatment. A decrease in β-d-glucan levels was observed over a few months in 45 patients who survived, in contrast to the high level of β-d-glucan in five patients who died. However, the serum β-d-glucan was not specific to *P. insidiosum* and therefore, all possible causes that affect β-d-glucan level should be ruled out to better understand the role of β-d-glucan.

### Histological examination

Standard histological stains, such as GMS and Periodic acid-Schiff (PAS) demonstrate the presence and extent of *P. insidiosum* in infected tissues (Krajaejun et al., 2006b; Keeratijarut et al., 2009; Mendoza, Prasla & Ajello, 2004). However, such stains may not differentiate *P. insidiosum* from other fungi (i.e., *Aspergillus* spp., *Fusarium* spp., Mucorales). Hematoxylin and eosin (H&E) staining shows tissue reactions, such as infiltration of eosinophils, purulent granuloma, and giant cells at the *P. insidiosum* infection site (Krajaejun et al., 2006b).

As opposed to the standard stains (i.e., GMS, PAS and H&E), several immunohistological staining assays have been developed to facilitate microscopic detection of *P. insidiosum* (Inkomlue et al., 2016; Keeratijarut et al., 2009; Triscott, Weedon & Cabana, 1993; Mendoza, Ajello & McGinnis, 1996; Mendoza, Kaufman & Standard, 1987; Brown et al., 1988). These assays rely on the use of rabbit antiserum generated against crude protein extract of *P. insidiosum*. For example, Mendoza et al. implemented a direct immunofluorescent assay using the rabbit anti-*P. insidiosum* antibodies conjugated with fluorescein isothiocyanate (Mendoza, Ajello & McGinnis, 1996; Mendoza, Kaufman & Standard, 1987). Brown et al. (1988), Triscott, Weedon & Cabana (1993), and Keeratijarut et al. (2009) introduced indirect immunochemical assays that require sequential staining reactions with rabbit anti-*P. insidiosum* antibodies (primary antibody), mouse or bovine anti-rabbit IgG (secondary antibody) tagged with peroxidase, and enzymatic substrates. These immunostaining assays require careful examination under a light microscope to confirm the presence of *P. insidiosum* in infected tissues. Interpretation of results may be complicated by false-positive staining of other fungi, such as *Conidiobolus* and *Fusarium* species (Keeratijarut et al., 2009; Grooters & Gee, 2002). Thus, confirmation of the *P. insidiosum* infection requires additional laboratory investigations. When fungal elements are histologically detected by GMS or PAS, a negative immunostaining result could exclude the possibility of *P. insidiosum*, due to the high sensitivity and negative predictive value of the tests (Keeratijarut et al., 2009).

A novel immunohistological method targeting elicitins that are only present in oomycetes, including *P. insidiosum* (Jiang et al., 2006; Lerksuthirat et al., 2015), has been reported to be highly specific. Recently, Inkomlue et al. used a rabbit antiserum against recombinant elicitin (ELI025) of *P. insidiosum* for the development of an immunohistochemical assay (Inkomlue et al., 2016), and found that all 38 *P. insidiosum* samples were detected, but not 49 control samples of various fungi, including *Fusarium* species and Mucorales*.*

### Molecular analysis

Conventional molecular techniques used to detect *P. insidiosum* are sequence homology analysis and PCR. The most popular target sequence is rDNA (also known as ribosomal RNA gene repeat). The rDNA contains 18S ribosomal RNA, internal transcribed spacer 1, 5.8S ribosomal RNA, internal transcribed spacer 2, and 28S ribosomal RNA (Grooters & Gee, 2002). These molecular techniques require a combination of universal fungal- or *P. insidiosum*-specific primers, template DNA extracted from pure culture or infected tissue, PCR reagents and equipment, and DNA sequencing facility (Badenoch et al., 2001; Vanittanakom et al., 2004; Znajda, Grooters & Marsella, 2002). Use of molecular techniques can substitute microbiological and immunological methods, especially when the latter fail to detect *P. insidiosum*. Badenoch et al. used universal fungal primers to amplify and sequence a DNA fragment from an organism isolated from a patient with an unknown ocular infection (Badenoch et al., 2001). Homology analysis of the obtained sequence (by BLAST searching against the GenBank database) identified the organism as *P. insidiosum*. Although the sequence homology analysis is a time-consuming and multi-step procedure, it is a standard method for the identification of many microorganisms, including *P. insidiosum*. One reason is that most reagents and tools required for this test are readily available in many clinical microbiology laboratories.

Using species-specific primers, PCR amplification can identify *P. insidiosum* from pure culture or infected tissue without DNA sequencing of the amplicon (Vanittanakom et al., 2004; Grooters & Gee, 2002; Znajda, Grooters & Marsella, 2002). Compared to sequence homology analysis, the PCR assay significantly reduces turnaround time from days to hours. Grooters et al. and Znajda et al. have co-developed a nested PCR assay, using the universal fungal primers (ITS1 and ITS2 or ITS2P) for the first-round reaction and the species-specific primers (PI-1 and PI-2) for the second-round reaction to specifically amplify *P. insidiosum* DNA (Grooters & Gee, 2002; Znajda, Grooters & Marsella, 2002). Vanittanakom et al. showed that the primers PI-1 and PI-2 (Grooters & Gee, 2002) failed to detect a Thai *P. insidiosum* strain. Therefore, a new set of *P. insidiosum*-specific primers (ITSpy1 and ITSpy2) was used in a simplified one round-PCR reaction (Vanittanakom et al., 2004) that was able to detect all four Thai strains tested. Thongsri et al. reported a single-tube nested PCR using a different set of primers (CPL6, CPR8, YTL1, and YTR1) for specific detection of *P. insidiosum* (Thongsri et al., 2013). More recently, Rujirawat et al. developed a multiplex PCR assay, using four primers (ITS1, Ra, R2, and R3) to target several single nucleotide polymorphisms of rDNA (Rujirawat et al., 2017). The multiplex PCR assay can simultaneously identify and genotype *P. insidiosum*.

Keeratijarut et al. showed that the rDNA-targeting primers, ITSpy1 and ITSpy2, were unable to PCR amplify certain *P. insidiosum* strains (Keeratijarut et al., 2014). They proposed the exo-1,3- β-glucanase-encoding gene (*exo* 1) of *P. insidiosum* as an alternative PCR target, and designed *exo* 1-specific primers, Dx3 and Dx4, for a conventional PCR assay. Based on a head-to-head comparison for PCR-based detection of 35 Thai *P. insidiosum* strains, the primers, Dx3 and Dx4, showed higher detection sensitivity than with ITSpy1 and ITSpy2 (100% vs. 91%) (Keeratijarut et al., 2014). Besides, the primers, Dx3 and Dx4, did not amplify non-specific target DNA from a variety of fungal species tested, showing 100% detection specificity. Keeratijarut et al. introduced an *exo* 1-targeting real-time PCR assay using the primers, Pr77 and Pr78, for detection of *P. insidiosum* (Keeratijarut et al., 2015). The detection sensitivity (100%) and specificity (100%) of the real-time PCR was high (Keeratijarut et al., 2015), but shortened the assay turnaround time by eliminating laborious and toxic steps (i.e., gel electrophoresis and ethidium bromide staining).

### Proteomic analysis

Matrix-assisted laser desorption ionization-time of flight mass spectrometry (MALDI-TOF MS) is a powerful diagnostic tool (Singhal et al., 2015; Sanguinetti & Posteraro, 2017; Jang & Kim, 2018) that can be applied for accurate identification of clinically-important microorganisms at low cost and short turnaround time. Unlike serological and molecular assays, MALDI-TOF MS does not require pathogen-specific reagents, such as primer, antigen, and antibody. Crude proteins of an unknown microorganism are extracted using an optimized protocol and subjected to the generation of main spectral profile (MSP) by MALDI-TOF MS analysis. The obtained MSP is searched against a reference database containing MSPs of many typed microorganisms. The matched organism with a significant score is, therefore, the identity of the unknown sample. Large mass spectrometric databases of a wide variety of microorganisms are currently available and accessible to assist the MALDI-TOF MS-based microbial identification (Singhal et al., 2015; Lau et al., 2013; Becker et al., 2014). Several mass spectrometric databases are now supplemented with the *P. insidiosum* MSPs aiding the identification of *P. insidiosum* (Bernheim et al., 2019; Krajaejun et al., 2018; Mani et al., 2019). The microorganism in question is reported as *P. insidiosum* if the MSP generated significantly matched its corresponding mass spectrum deposited in the reference database.

## Management and Follow-up

The treatment of pythiosis can be categorized into surgical intervention, medications (antimicrobial and related agents), and immunotherapy. Based on the experiences of our center and that of other groups, *P. insidiosum* is usually resistant to both systemic and traditional topical antifungal agents. Since aggressive medical treatment often fails to cure the infection, prompt surgical intervention is the key treatment to control the disease. Potential therapeutic options for patients with vascular and ocular pythiosis are presented below:

### Vascular pythiosis

#### Presurgical assessment

Urgent preoperative assessment determined by computerized tomography angiogram (CTA) of affected vessel (i.e., lower extremities) up to the proximal origin of vessels (i.e., CTA of whole aorta with femoral run off) is recommended. Abnormal radiography showing thickening of the vessel wall with enhancement, thrombosis, and aneurysmal dilation may indicate arteritis (Krajaejun et al., 2006b; Sermsathanasawadi et al., 2016; Chitasombat et al., 2018b). The CTA method has some advantages compared with angiogram for the determination of soft tissue and lymph node involvement. Magnetic resonance imaging with angiogram (MRI/MRA) is the preferred modality to assess cranial involvement, i.e., septic emboli, small abscesses, and cavernous sinus extensions (Rathi et al., 2018; Chitasombat et al., 2018b; Narkwiboonwong et al., 2011).

#### Surgical intervention

Prompt radical surgery leading to amputation to achieve pathogen-free surgical margin is the mainstay of successful treatment of vascular pythiosis (Krajaejun et al., 2006b; Worasilchai et al., 2018; Sermsathanasawadi et al., 2016). The suggested adequate resected proximal margin is 5 cm above the site of the arterial lesion detected by CTA (Sermsathanasawadi et al., 2016; Sangruchi et al., 2013). Prior to surgery, scheduled arrangement with pathologist ensures immediate intraoperative results to enable surgical decisions. Intraoperative soft tissue and vascular margin should be assessed carefully for macroscopic (gross appearance) and microscopic examination with real-time frozen section for KOH stain (Sermsathanasawadi et al., 2016). Intraoperative frozen section specimen is crucial to determine the free surgical margin (Chitasombat et al., 2018a; Sermsathanasawadi et al., 2016). The organism-positive margin required re-excision at ≥ 5 cm proximally, and re-examination by KOH test until the arterial margins and surrounding soft tissue margins were free of disease (Sermsathanasawadi et al., 2016). Inadequate assessment of surgical-free margin at the time of surgery leads to progression of residual disease involving the proximal artery, i.e., aorta (Chitasombat et al., 2018a). Some patients require repeated aggressive surgical procedure, i.e., below/above knee amputation, hip disarticulation, hemipelvectomy, and aneurysmectomy (Permpalung et al., 2015). Patients with common iliac artery or aortic involvement who underwent aneurysmectomy with bypass grafting survived only few months (Krajaejun et al., 2006b; Permpalung et al., 2015; Sathapatayavongs et al., 1989; Sermsathanasawadi et al., 2016; Wanachiwanawin et al., 1993).

#### Antifungal drugs

Antifungals are generally ineffective against *P. insidiosum* as they do not target enzymes in the ergosterol biosynthetic pathway (Lerksuthirat et al., 2017). This confers clinical resistance to antifungals, which could only be effective at the difficult-to-achieve concentrations (Lerksuthirat et al., 2017). *In vitro* minimal inhibitory concentration (MIC) data revealed amphotericin B had the highest MIC, followed in order by voriconazole, fluconazole, anidulafungin, caspofungin, itraconazole, and terbinafine (Permpalung et al., 2015). Combination of itraconazole and terbinafine was commonly used, owing to its ability to synergistically inhibit growth *in vitro* (Krajaejun et al., 2006b; Shenep et al., 1998; Kirzhner et al., 2015; Argenta et al., 2008). However, synergistic effects between itraconazole or voriconazole and terbinafine could not be demonstrated in Thai *P. insidiosum* isolates (Permpalung et al., 2015; Worasilchai et al., 2018).

Prolonged treatment with a combination of itraconazole and terbinafine successfully cured a few patients with unresectable disease (Krajaejun et al., 2006b; Shenep et al., 1998). The *in vitro* activity of various antifungals were explored; terbinafine combined with caspofungin or fluconazole showed synergistic activity in Brazilian isolates (Cavalheiro et al., 2009). Susceptibility should be cautiously interpreted as there are several methods of antifungal susceptibility determination without standardized assays and regional differences exist for various strains/genotypes (Permpalung et al., 2015; Lerksuthirat et al., 2017; Schurko et al., 2003a; Schurko et al., 2003b). New triazoles, i.e., voriconazole and posaconazole, have been used to successfully treat patients with residual unresectable disease (Kirzhner et al., 2015). Recent *in vitro* data revealed that 12.5% of Thai *P. insidiosum* strains had voriconazole MIC >4 mg/l, whereas approximately 70–80% of isolates had itraconazole MIC >1 mg/l (Permpalung et al., 2015; Worasilchai et al., 2018; Permpalung et al., 2019; Susaengrat et al., 2019). Regarding the duration of treatment, combination of itraconazole and terbinafine treatment for 1–2 years has been recommended based on a successful outcome of unresectable vascular pythiosis (Worasilchai et al., 2018; Shenep et al., 1998; Sermsathanasawadi et al., 2016). However, treatment protocols should be tailored to the individual patient’s needs according to clinical, laboratory data, and radiographic information (see ‘follow-up and monitoring’).

#### Antimicrobial agents

Historically, saturated solution of potassium iodide (SSKI; one mL orally three times per day for up to three months) has been used to successfully treat localized skin/subcutaneous disease (Krajaejun et al., 2006b; Imwidthaya, 1994). Several antibacterial drugs that inhibit protein synthesis can inhibit the growth of *P. insidiosum* (Brazilian strains) *in vitro* including azithromycin, tigecycline, clarithromycin, linezolid, nitrofurantoin, chloramphenicol, quinupristin/dalfopristin, clindamycin, josamycin, miltefosine, sutezolid, retapamulin, tiamulin, and valnemulin (Loreto et al., 2011; Mahl et al., 2012; Jesus et al., 2014; Jesus et al., 2016; Itaqui et al., 2016; Loreto et al., 2019). Azithromycin demonstrated potent *in vivo* activity in an experimental model of pythiosis (Jesus et al., 2016; Loreto et al., 2018). In a recent study, azithromycin, doxycycline, and clarithromycin combined with itraconazole or voriconazole were administered as salvage therapy in two patients with relapsed vascular pythiosis (aortic aneurysm), in whom surgery cannot be performed (Susaengrat et al., 2019). Both patients responded well to combination of doxycycline and clarithromycin or azithromycin and survived up to 64 weeks of follow-up (Susaengrat et al., 2019)**.** *In vitro* susceptibility of *P. insidiosum* isolates revealed clarithromycin, azithromycin, minocycline, and doxycycline MICs of 0.5, 2, 2, and 4 mg/l, respectively. Furthermore, azithromycin or clarithromycin combined with either minocycline or doxycycline had *in vitro* synergistic effect (Susaengrat et al., 2019).

#### Iron chelators

Human pythiosis was first described in a thalassemia patient in 1989 (Sathapatayavongs et al., 1989). Since then, vascular pythiosis mainly affects thalassemia patients and iron overload has been associated with the pathogenesis of pythiosis (Krajaejun et al., 2006b). *P. insidiosum* possesses the gene encoding ferrochelatase, which indicates role of iron in the pathogenesis (Krajaejun et al., 2011). Iron overload is known to alter T and B cell proliferation (Schaible & Kaufmann, 2004) because of which, patients with thalassemia are susceptible to infection owing to impaired immune responses in monocytes/macrophages and cytokine production (Ud-Naen et al., 2019; Wanachiwanawin et al., 1993). Iron chelation augments tumor necrosis factor alpha (TNF-α), GM-CSF and IFN-γ release from monocytes/macrophages in thalassemia patients irrespective of ferritin levels (Ud-Naen et al., 2019). *In vitro* data showed that deferasirox directly damages *P. insidiosum* hyphae (Zanette et al., 2015). In the rabbit model of pythiosis, deferasirox exhibits an immuno-modulating effect which is similar to that of the immunotherapy (Zanette et al., 2015). Clinically, various iron chelators, i.e., deferiprone, deferasirox, and deferoxamine have been used adjunctively in thalassemia patients to treat iron overload (Permpalung et al., 2015; Worasilchai et al., 2018). Iron chelation therapy did not appear to change the treatment outcome in vascular pythiosis (Permpalung et al., 2015).

#### Immunotherapy

This strategy was first used successfully in 1998 as an adjunct treatment in patients with unresectable disease (Khunkhet, Rattanakaemakorn & Rajatanavin, 2015). The PIA vaccine, derived from endoplasmic and secretory antigens of the pathogen (Krajaejun et al., 2006b), shifts immune response from the T-helper 2 to T-helper 1 for cytotoxic killing of hyphae (Wanachiwanawin et al., 2004). The vaccine demonstrated an acceptable safety profile, but the efficacy remains inconclusive due to the small sample size (Permpalung et al., 2015; Wanachiwanawin et al., 2004). Only a few patients with inoperable disease survived (Khunkhet, Rattanakaemakorn & Rajatanavin, 2015; Susaengrat et al., 2019). Adverse reaction to the vaccine included local swelling, redness, pruritus, minor rash, and regional lymphadenopathy (Permpalung et al., 2015; Wanachiwanawin et al., 2004). A severe inflammatory reaction at the site of infection showing a massive periorbital/facial swelling resulting in respiratory distress was reported in a pediatric patient from the United States after the third dose of vaccine (Kirzhner et al., 2015). Nowadays in Thailand, PIA vaccine is administered as an adjunct treatment (Krajaejun et al., 2006b; Worasilchai et al., 2018; Sermsathanasawadi et al., 2016). Various PIA preparations derived from protein antigen prepared from *P. insidiosum* have been used (Permpalung et al., 2015; Sermsathanasawadi et al., 2016; Wanachiwanawin et al., 2004; Mendoza, Mandy & Glass, 2003). The vaccination schedules varies across different institutions in Thailand (Permpalung et al., 2015; Sermsathanasawadi et al., 2016). The commonly administered schedule in Thailand is vaccine preparations of one milliliter of 2 mg/ml PIA given subcutaneously at diagnosis and at 0.5, 1, 1.5, 3, 6, and 12 months (Worasilchai et al., 2018). Host response to PIA vaccine showed inter-individual variations. The measurement of *P. insidiosum*-specific antibody titer by ELISA can be used to monitor host immune to PIA vaccine; patient with higher titer in the absence of active ongoing infection had survival advantage (Worasilchai et al., 2018).

#### Follow-up and monitoring

Daily physical assessment of soft tissue, surgical sites, lymphadenopathy and signs of vascular insufficiency are critical after surgery. Stump abscesses, and myositis along with evidence of arterial insufficiency syndrome (arteritis, thrombosis, aneurysm, pulsatile mass) represent residual disease that necessitates investigation and aggressive management (Chitasombat et al., 2018a). Neutropenic patients should be monitored closely due to rapid clinical deterioration, i.e., progression of cellulitis (Chitasombat et al., 2018a). Early postoperative CTA may show non-specific findings, i.e., swelling, and thrombosis of stump at the ligated vessel (Chitasombat et al., 2018a). Follow-up imaging should be performed if disease relapse is suspected, which can occur several months after surgery (Susaengrat et al., 2019). Arteritis should be suspected when certain radiographic clues, such as thickening of the vessel wall with contrast enhancement, thrombosis, and aneurysmal dilation, are observed (Chitasombat et al., 2018a; Sermsathanasawadi et al., 2016). Patients with disease involving iliac vessel or femoral artery may relapse, despite the negative surgical margin, given that the vessels are in the proximity of aorta (Susaengrat et al., 2019).

Serum β-d-glucan and *P. insidiosum*-specific antibody are potential biomarkers of vascular pythiosis after treatment initiation (Worasilchai et al., 2018; Susaengrat et al., 2019). Serum β-d-glucan declined three months after surgery and became undetectable among survivors (Worasilchai et al., 2018). Persistently elevated serum β-d-glucan at two weeks after surgery should prompt an evaluation for residual disease (Worasilchai et al., 2018). Furthermore, an elevated trend of *β*-d-glucan from baseline should prompt an investigation and treatment for disease relapse (Susaengrat et al., 2019). Death patients had statistically significant higher levels of serum β-d-glucan, compared with survivors (Worasilchai et al., 2018).

A robust host immune response to PIA vaccine is demonstrated by *P. insidiosum*-specific antibody titer level. A high level of *P. insidiosum*-specific antibody measured by ELISA after PIA vaccine administration shows a statistically significant association with survival (Worasilchai et al., 2018).

### Ocular pythiosis

#### Surgical intervention

Early therapeutic penetrating keratoplasty with at least one mm clear margin is considered the gold standard for eradicating *Pythium* keratitis. Radiating reticular pattern must be included within the keratoplasty. Complete elimination of the exudate from the anterior chamber prevents recurrence of infection. Patients with global salvage underwent the first surgical excision earlier than patients who eventually lost their eyes (Permpalung et al., 2019). However, in eyes with very large or peripheral lesions extending up to the limbus, the outcome of therapeutic penetrating keratoplasty may be poor with a high risk of recurrence, because the surgical margin may not be free of infection. Adjunct therapy is therefore recommended in such cases to reduce the risk of recurrence (Agarwal et al., 2019; Agarwal et al., 2018). Application of single freeze-thaw cryotherapy on the host at the graft-host junction or at the limbus using 2 mm-tip retinal cryoprobe for 7–8 s has been used successfully to prevent recurrence (Agarwal et al., 2018). Additionally, in patients with scleral involvement, 99.9% absolute alcohol should be applied over and beyond the area of cryotherapy application extending to the posterior edge of infected sclera for 20 s to reduce the potential detrimental effects of multiple rows of cryotherapy (Agarwal et al., 2018). To avoid delays in surgery, glycerin-preserved corneal grafts or scleral grafts are alternatives to corneal grafts when corneal donor tissues are not available (Thanathanee et al., 2013). Evisceration or enucleation may be required in cases of extensive lesions involving large area of sclera or endophthalmitis (Lekhanont et al., 2009; Neufeld et al., 2018).

#### Antimicrobial agents

As mentioned earlier (see vascular pythiosis section), *P. insidiosum* is generally refractory to antifungals, which reflects the failure of medical treatment in majority of cases. However, few cases were resolved with topical 1% itraconazole and only one eye of a patient with bilateral keratitis responded to medical therapy (0.15% amphotericin B, 2% ketoconazole, 1% voriconazole, and oral terbinafine) (Anutarapongpan et al., 2018; Hasika et al., 2019).

Despite the lack of management strategies for *Pythium* keratitis, recent advances in therapeutics are encouraging. Several studies showed *in vitro* susceptibility of various strains of *P. insidiosum* to antibacterial agents that inhibit protein synthesis including tigecycline, macrolides, tetracyclines, and linezolid, either as monotherapy or as adjunct therapy to antifungal agents (Mahl et al., 2012; Jesus et al., 2014; Jesus et al., 2016; Loreto et al., 2014). A large ocular series showed *in vitro* activity of tigecycline, mupirocin, and minocycline against Indian *P. insidiosum* strains (Bagga et al., 2018). The first patient with presumptive *Pythium* keratitis was successfully managed non-surgically with a triple regimen, consisting of topical 0.2% linezolid every hour, topical 1% azithromycin every 2 h, and oral azithromycin 500 mg once daily for 3 days a week (Ramappa et al., 2017). One presumptive case and two confirmed cases of *Pythium* keratitis were successfully treated with similar regimens, including a combination of topical and oral azithromycin and topical voriconazole, a combination of topical minocycline and chloramphenicol and oral linezolid, and a combination of oral doxycycline and clindamycin and topical azithromycin, respectively (Chatterjee & Agrawal, 2018; Maeno et al., 2019; Bernheim et al., 2019). Intracameral injection and oral minocycline have also been used along with repeated keratoplasty to successfully treat a patient with recurrent infection after the second corneal transplant (Ros Castellar et al., 2017). A favorable, but not statistically significant, response of *Pythium* keratitis to a triple combination of topical linezolid and topical and oral azithromycin was found in a large pilot series of 18 patients (Bagga et al., 2018). The percentage of success and failure in smaller lesions (≤ 6 mm) was approximately equal and there was 100% failure in eyes with larger lesions (>6 mm) (Bagga et al., 2018). Furthermore, all patients in another series failed to respond and/or worsened with the triple regimen (Agarwal et al., 2019). Additional clinical trials are needed to investigate the true efficacy of these antibiotics, which have a common mechanism of action —inhibition of protein synthesis. Thus, treatment may be considered an adjuvant therapy after surgical excision.

#### Immunotherapy

For some patients with ocular pythiosis, PIA has been used together with other therapeutic modalities as compassionate therapy (Lekhanont et al., 2009; Permpalung et al., 2015; Permpalung et al., 2019; Thanathanee et al., 2013). Three patients received subcutaneous administration of PIA to prevent post-keratoplasty recurrence in corneal grafts (Lekhanont et al., 2009; Thanathanee et al., 2013). Although no recurrence was seen in two of three patients after re-graft, combined with three doses of vaccine, this may be attributed to keratoplasty with a wide surgical excision. The other two series reported the use of PIA at diagnosis and showed that 43–47% of patients needed evisceration/enucleation to be cured of the disease (Permpalung et al., 2015; Permpalung et al., 2019). It is interesting to note that the use of PIA has been reported solely from Thailand. To be declared effective, larger, well-designed studies on the efficacy and safety of PIA are necessary.

#### Follow-up and monitoring

Slit-lamp examination of *Pythium* keratitis patients may demonstrate a remarkable increase in corneal infiltration in a day (Lekhanont et al., 2009). Therefore, daily clinical monitoring is highly recommended. Recurrence after therapeutic penetrating keratoplasty could be as high as 45–75% of cases (Anutarapongpan et al., 2018; Agarwal et al., 2019; Thanathanee et al., 2013; Agarwal et al., 2018), and occurs within the first two weeks after surgery (Lekhanont et al., 2009; Thanathanee et al., 2013). The graft-host junction and the anterior chamber should be carefully inspected for new infiltration or newly formed white exudate inside the chamber (Agarwal et al., 2019). If the warning signs are present, prompt re-grafting and adjunct therapies should be considered to avoid eye loss. Extensive infection involving the sclera and extraocular muscles could potentially lead to cavernous sinus thrombophlebitis (Rathi et al., 2018; Chitasombat et al., 2018b). Thus, imaging study of brain and orbit should be considered to determine disease extension and treatment planning.

### Clinical outcomes

#### Vascular pythiosis

The mortality rate of vascular pythiosis ranged between 31% and 100% (Krajaejun et al., 2006b; Permpalung et al., 2015; Chitasombat et al., 2018a; Sermsathanasawadi et al., 2016). The main predictor of survival was the microscopic demonstration of *P. insidiosum*-free surgical margin (vascular and soft tissue) (Sermsathanasawadi et al., 2016). Disease recognition is an important measure for pre-surgical diagnosis, adequate surgery, and tissue diagnosis (Chitasombat et al., 2018a). Delay in diagnosis and surgical treatment contributed to advanced disease which was beyond surgical cure (Permpalung et al., 2015; Reanpang et al., 2015; Sermsathanasawadi et al., 2016). A recent prospective cohort study explored the use of new monitoring tools, including serum β-d-glucan and *P. insidiosum*-specific antibody, and showed that the mortality rate reduced to 10%, given that most patients had free surgical margin (Worasilchai et al., 2018). Patients with unresectable disease involving the common iliac artery and aorta often did not survive (Krajaejun et al., 2006b; Permpalung et al., 2015; Sermsathanasawadi et al., 2016). There are few reports of patients with suprainguinal vascular pythiosis who survived after aggressive surgical eradication, i.e., amputation and surgical removal of all infected arteries, extending to the common iliac artery, in conjunction with antifungal agent, and PIA immunotherapy (Hahtapornsawan et al., 2014). Recently, new therapeutic modalities comprising a combination of antifungals, adjunct antimicrobials and PIA vaccine, along with surgery successfully stabilized disease in two patients up to 64 weeks of follow-up (Susaengrat et al., 2019). Overall survival depends on several factors; site of vascular involvement, duration of symptoms to the first surgery, definitive surgery, negative surgical margin, underlying disease, immune response to PIA vaccine.

#### Ocular pythiosis

In the past, up to 90% of the cases required evisceration or enucleation (Krajaejun et al., 2004; Kunavisarut, Nimvorapan & Methasiri, 2003). Recently, a large case series of 46 eyes with *Pythium* keratitis demonstrated that the rate of recurrence after penetrating keratoplasty was 55% and the evisceration rate was reduced to 15% of the cases (Agarwal et al., 2019). None of the cases recurred following therapeutic keratoplasty with adjunct procedures and enucleation was not needed (Agarwal et al., 2019). Available data show that experienced clinicians who promptly perform surgery and provide adjunct measures including cryotherapy and absolute alcohol treatment, enable favorable treatment outcome.

## Conclusions

Human pythiosis is a life-threatening condition with high morbidity and mortality. Vascular and ocular pythiosis are common clinical manifestations. Early diagnosis and timely intervention are the keys to an optimal outcome. Although diligent records of patient medical history and physical examination provide diagnostic clues on “pythiosis”, definitive disease diagnosis requires laboratory testing. In addition to traditional microbiological methods, diagnostic assays (i.e., serological, molecular, and proteomic tests) have been developed to aid diagnosis of pythiosis. Selection of the diagnostic tests relies on assay availability, detection efficiency, and experience of laboratory personnel. Regarding the management of vascular pythiosis, surgical intervention that achieves a *Pythium*-free margin of the affected tissue, in combination with the administration of antifungal drugs and PIA, remain the recommended treatment. Use of adjunct antimicrobials holds considerable promise in mitigating disease relapse. During treatment, clinical assessment of the vascular pythiosis patient should be performed daily. Ocular pythiosis is a critical ophthalmological condition where a high degree of suspicion and precise recognition of the typical clinical features of ocular pythiosis are warranted for early diagnosis. While the role of therapy for ocular pythiosis (i.e., antimicrobial drugs and PIA) remains uncertain, early therapeutic penetrating keratoplasty with clear surgical margins is the gold standard for achieving global salvage. To prevent recurrence after eye surgery, adjunct strategies including perioperative cryotherapy and absolute alcohol application may be beneficial.

## Additional Information and Declarations

### Data Availability

The following information was supplied regarding data availability: The study (review article) generated no data nor code.
